# A Novel Two-Tier Cooperative Caching Mechanism for the Optimization of Multi-Attribute Periodic Queries in Wireless Sensor Networks

**DOI:** 10.3390/s150715033

**Published:** 2015-06-26

**Authors:** ZhangBing Zhou, Deng Zhao, Lei Shu, Kim-Fung Tsang

**Affiliations:** 1 School of Information Engineering, China University of Geosciences (Beijing), Beijing 100083, China; E-Mail: zhaodpx@163.com; 2 Guangdong Provincial Key Lab. of Petrochemical Equipment Fault Diagnosis, Guangdong University of Petrochemical Technology, Maoming 525000, China; E-Mail: lei.shu@ieee.org; 3 Department of Electronic Engineering, City University of Hong Kong, Hong Kong, China; E-Mail: ee330015@cityu.edu.hk; 4 Computer Science Department, TELECOM SudParis, Evry 91011, France

**Keywords:** periodic query optimization, cooperative caching, wireless sensor networks

## Abstract

Wireless sensor networks, serving as an important interface between physical environments and computational systems, have been used extensively for supporting domain applications, where multiple-attribute sensory data are queried from the network continuously and periodically. Usually, certain sensory data may not vary significantly within a certain time duration for certain applications. In this setting, sensory data gathered at a certain time slot can be used for answering concurrent queries and may be reused for answering the forthcoming queries when the variation of these data is within a certain threshold. To address this challenge, a popularity-based cooperative caching mechanism is proposed in this article, where the popularity of sensory data is calculated according to the queries issued in recent time slots. This popularity reflects the possibility that sensory data are interested in the forthcoming queries. Generally, sensory data with the highest popularity are cached at the sink node, while sensory data that may not be interested in the forthcoming queries are cached in the head nodes of divided grid cells. Leveraging these cooperatively cached sensory data, queries are answered through composing these two-tier cached data. Experimental evaluation shows that this approach can reduce the network communication cost significantly and increase the network capability.

## 1. Introduction

With the rapid development of microelectronic, wireless communication, new and renewable energy technologies, smart sensor nodes become smaller in physical size, stronger in storage and computational capabilities, more powerful in battery capacity and less expensive in price. Sensor nodes form wireless sensor networks (WSNs), which have been adopted in widespread domain applications, including ambient assisted living [1], target tracking [2], bridge or traffic monitoring [3], *etc.* Sensor nodes are mostly battery-powered, which are difficult to be recharged and replaced, especially when deployed in harsh environments. Although energy harvesting from natural sources [4], energy replenishment [5,6] and radio optimization and charging [7,8] technologies have been developed to recharge the battery of sensor nodes, network lifetime maximization and prolongation is still essential, and energy efficiency is one of the most important research challenges in WSNs nowadays [7]. Note that the network lifetime can be defined as various semantics depending on the context of application domains, and a definition with wide acceptance is the time when the first sensor node depletes its energy [9]. Therefore, techniques that facilitate sensory data gathering efficiently for answering queries, while prolonging the network lifetime as much as possible, are fundamental.

As presented by Xu *et al.* [10], queries in WSNs are typically conducted in a periodic, rather than one-shot, fashion for supporting one or multiple applications. Note that queries in WSNs are different from those in query-based WSNs [11], where sensor nodes are producers (sources) and consumers (sinks) of resources simultaneously. In this article, sensor nodes gather sensory data, which are aggregated and routed to the sink node according to the requirement of certain applications. Usually, WSNs can be shared by multiple applications to improve the network utilization efficiency [12]. Consequently, multiple queries are performed in a certain time period, and multiple-attribute sensory data are often interested [13,14]. These queries may have overlapping sub-regions of interest. Besides, the points of interest for certain applications may be within a certain sub-region for a certain time duration, while evolving moderately to a neighboring sub-region [15,16]. In this setting, when the number of queries is relatively large and each query is to be processed independently, the capability required for processing these queries may be above the capability that the network can provide, and consequently, the delay for query answering may be towards infinity [10,17]. Therefore, the mechanism for the optimization of multi-attribute query processing, while prolonging the network lifetime, is an important research challenge.

Traditional techniques have studied the query processing in WSNs from various aspects, including in-network query processing [18], aggregated query processing [19], compressed data aggregation [20], spatial correlation data aggregation [21], range query processing [22], opportunistic sampling-based query processing [23], snapshot and continuous data aggregation [17,24], real-time query processing [25], multiple dimensional or attributes query optimization [26–28], cooperative caching-based query processing [29,30], *etc.* Generally, these techniques are mostly exploring the one-shot query scheduling, where one single attribute is interested, whereas few efforts study periodic, aggregated and multi-attribute query processing [31]. Note that queries to be conducted in a certain time duration usually have overlapping sub-regions, where sensory data in these sub-regions gathered from the network can be shared for answering these concurrent queries. Besides, sensory data gathered at the current time slot may be reused for answering the forthcoming queries, when the variation of these sensory data is within an allowed threshold. In fact, sensory data may not vary dramatically in certain applications (like health or environmental monitoring), and many applications may work well when the bias of sensory data ((i) being used and (ii) being sensed in real time) is within a certain threshold [32]. Without loss of generality, a time duration is divided and represented by discrete time slots. Queries issued at a certain time slot are rewritten into one query. Hence, these concurrent queries are processed in batches. Besides, certain sensory data are cached in the network for answering the forthcoming queries and are retrieved from the network when the bias between the cached value and the current sensing value is above a certain threshold. Generally, this threshold is pre-specified as an appropriate value considering the characteristics of certain attributes and the requirements of certain applications. This strategy should reduce the network communication cost, improve the network capability, shorten the response time of query answering and, most importantly, prolong the network lifetime to some extent. Consequently, caching and refreshing multi-attribute sensory data in the network, while diminishing the cost of answering the forthcoming queries, is an important research problem to be explored further.

To remedy this issue, a two-tier popularity-based cooperative caching (PCC) mechanism is developed to support the periodic query processing, where multi-attribute sensory data are cached in the sink node and leaf head nodes of an index tree. Our main contributions are presented as follows:

- Given a network represented as square grid cells with inverted files, an index tree is constructed, where grid cells correspond to the leaf nodes in this tree. A query expects to return sensory data for certain attributes in a set of neighboring grid cells. For simplicity, sensory data in a grid cell with an attribute is considered as an atomic unit for query answering and caching manipulation. A query is answered through composing sensory data that are: (i) cached in the sink node; and (ii) gathered from the network in real time.
- The sink node is usually limited in storage and computational capabilities and can hardly cache sensory data of all sensor nodes in the whole network. Besides, a sub-region, rather than the whole network, is usually interested in applications within a certain time duration. Therefore, a two-tier cooperative caching mechanism is proposed, such that sensory data of the most popular are cached at the sink node, and these data can be reused for answering the forthcoming queries. This strategy can reduce the energy consumption of answering concurrent queries to a large extent. Specifically, the popularity of sensory data, which are interested in most queries in the most recent time slots, is the highest. These sensory data are assumed to be interested mostly in the forthcoming queries and are cached in the sink node. On the other hand, as for a grid cell that may not be interested in queries at this moment, sensory data in this grid cell are cached locally in the memory space of the corresponding head node. A flag, which indicates that sensory data have been varied significantly, is cached in the head node. When this grid cell is interested in queries, sensory data of these sensor nodes, whose flags indicate a dramatic variation, are retrieved from the network in real time.
- Extensive simulations are conducted for evaluating the effectiveness and efficiency of the proposed algorithms. The experimental results show that the technique proposed in this article outperforms another technique proposed by Zhou *et al.* [33] where multiple-attribute query processing is also explored. Generally, our technique is more efficient than [33] in reducing the communication energy consumption and increasing the network capability, especially when the number of queries and the number of attributes interested in these queries are relatively large.

The rest of this article is organized as follows. Section 2 introduces the energy model used in the following. Section 3 presents the index tree construction algorithm and the network caching model. Section 4 proposes our cooperative caching mechanism for facilitating query answering. Section 5 evaluates the technique developed in the previous sections. Section 6 reviews and compares traditional techniques, and Section 7 concludes this work.

## 2. Preliminary: Energy Model

Several protocols for wireless sensor networks have been proposed leveraging the assumption made for the radio characteristics in the transmission and receiving modes. Without loss of generality, we apply the well-adopted first order radio model [34] in this article, for the computation of the energy consumption, where the parameters are presented in Table 1. In this model, the energy consumed for running the transmitter or receiver circuitry *E_elec_* is set to 50 nJ/bit, while that for the transmit amplifier *ϵ_amp_* is set to 100 pJ/bit/m^2^. The energy consumption(s) *E_Tx_*(*k, d*) (or *E_Rx_*(*k*)) for transmitting (or receiving) a packet of *k* bits within a distance *d* is (are) specified by the following equations:

(1) $$E_{Tx}\left(k,d\right)=E_{\text{elec}}\times k+\in_{\text{amp}}\times k\times d^{n}$$

(2) $$E_{Rx}\left(k\right)=E_{\text{elec}}\times k$$

Consequently, the energy consumption *E_ij_*(*k*) for transmitting a packet of *k* bits from a sensor node *i* to a neighboring sensor node *j* is computed as follows:

(3) $$E_{ij}\left(k\right)=E_{Tx}\left(k,d\right)+E_{Rx}\left(k\right)=\{\begin{array}{cc} E_{\text{elec}}\times k+\in_{\text{amp}}\times k\times d^{n} & \text{if}\;j\;\text{is}\;SN\\ 2\times E_{elec}\times k+\in_{amp}\times k\times d^{n} & \text{otherwise} \end{array}$$

Note that the energy consumption of transmitting a packet to a sensor node is different from that to the sink node (SN, or called the base station), since SN is assumed to have no energy constraint, and the cost of receiving a packet is ignored in this model [34]. The parameter *d* refers to the distance between sensor nodes *i* and *j* (or SN). The energy of transmitting a packet from a sensor node *i* to another *j* is assumed the same as that of transmitting a packet from *j* to *i*, *i.e.*, *E_ij_*(*k*) = *E_ji_*(*k*). The parameter *n*, which refers to the attenuation index of transmission as presented in Table 1, is determined by the surrounding environment. If sensor nodes in the network are barrier-free when forwarding packets, *n* is set to two. Otherwise, *n* is set to a value between three and five, when sensor nodes for long-distance transmission are distributed in the area of buildings and a vegetation cover. Without loss of generality, the network is assumed to be deployed in an area that is barrier-free, and *n* is set to two in our experiments in Section 5.

**Figure 1:**
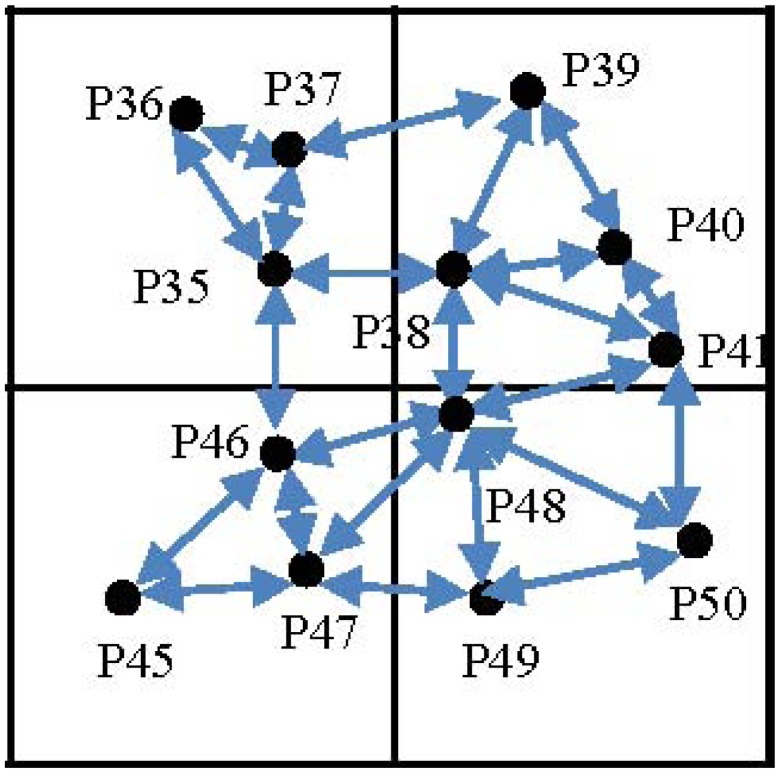
An example of energy consumption for the transmission of packets, where the sub-region is part of the network region, as shown in Figure 2.

**Figure 2:**
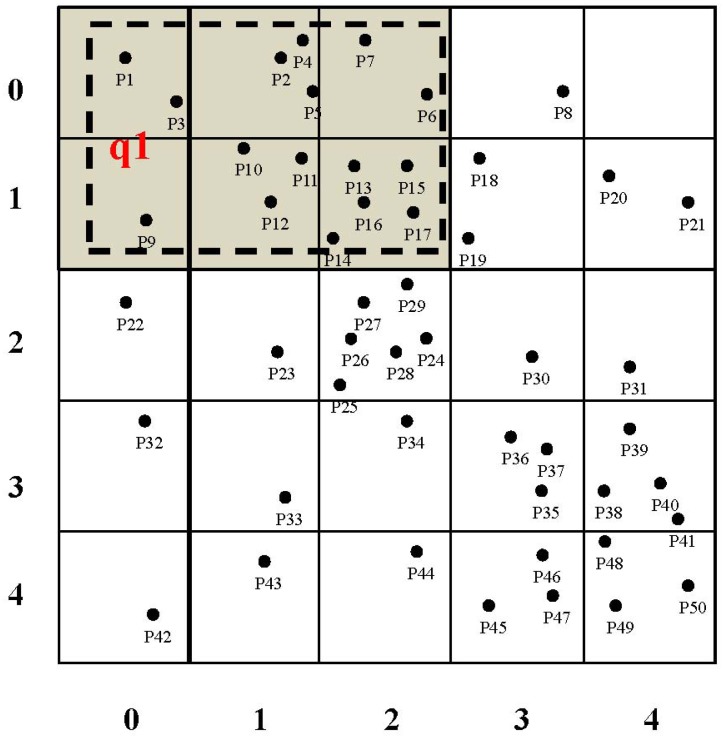
An example of grid division, where 50 sensor nodes are deployed unevenly in the network region and several kinds of attributes are assumed sensed by these sensor nodes. The network region is divided into 25 square grid cells, which are the same in geographical size. The region of a query (for instance, *q*1) is rewritten into a set of grid cells. For instance, *q*1.*q*r can be rewritten into a set of grid cells of {*gc*_0_, *gc*_1_, *gc*_2_, *gc*_5_, *gc*_6_, *gc*_7_}.

As an example, Figure 1 shows part of our sample network region, as shown in Figure 2, where 13 sensor nodes are deployed in this sub-region. The lines of arrows reflect the fact that sensor nodes in neighboring grid cells are within their communication radius r. Consequently, the energy consumed for forwarding a packet with the size of k bits from a sensor node (e.g., 47) to a neighboring one (e.g., 49) is computed as *E_ij_*(*k*) = 2 × *E_elec_* × *k* + *ε_amp_* ×*k* × *d^n^* = 2×50×*k* + 0.1 × *k* × *d*^2^, where the parameter *d* represents the geographical distance between sensor Nodes 47 and 49.

## 3. Index Tree Construction and Network Caching Model

This section proposes an index tree construction algorithm leveraging the method developed by Zhou *et al.* [33] and introduces the network caching model, which is used to facilitate the query processing in Section 4.

### 3.1. Index Tree Construction

A tree-based routing structure has been used widely in WSNs for supporting the data gathering, aggregation and transmission in a multi-hop manner [35,36]. Leveraging the index tree construction algorithm developed in our previous work [33], we develop a novel index tree for organizing sensor nodes in a balanced manner, which is better at facilitating the query processing when multiple kinds of attributes are interested according to certain requirements of domain applications. Similar to the assumptions made in our previous work [33], sensor nodes are assumed in a skewed distribution, where sensor nodes are dense in some sub-regions of the network, while they are sparse in the others. In fact, sensor nodes are distributed unevenly in real-world applications, including bridge or traffic monitoring scenarios [3]. An example of sensor nodes in a skewed distribution is shown in Figure 2, where sensor nodes located in the upper center and the right bottom of the network region are dense, while the others are sparse. A sensor node is assumed accompanied by one sensing equipment for sensing a certain attribute, such as humidity, temperature, gas flow, *etc.* Various sensor nodes with different sensing equipment are able to sense diverse attributes. The index tree construction algorithm proposed by Algorithm 1 in our previous work [33] works as follows:

- The network region is divided into square grid cells whose side-length is set to $\sqrt{2r}$ , and an inverted file [37] is attached to each grid cell for specifying the attributes to be sensed by the sensor nodes therein. An example of grid cell division is shown in Figure 2, where the network is divided into 25 grid cells, and a two-dimensional matrix is adopted to represent the coordinates of these grid cells. Actually, grid cells correspond to the leaf nodes of the index tree to be constructed.
- The weight between two neighboring grid cells or sub-regions is calculated according to the mechanism proposed by the Algorithm 2 in our previous work [33]. This weight specifies the energy consumption of forwarding the same size of message between neighboring grid cells. Intuitively, sensor nodes in neighboring grid cells contribute to this weight calculation when the Euclidean distance between them is no more than the communication radius of sensor nodes *r*.
- Neighboring grid cells or sub-regions are merged in a pair-wise fashion when the weight between the candidates is the greatest, and the inverted file is processed accordingly. This merging procedure iterates until the root node of the index tree, which is a binary tree in shape, is constructed. Note that this merging strategy makes adjacent sub-regions, which may induce relatively larger energy consumption when forwarding messages in between, to be included by different children. Consequently, the energy consumption for routing data packets along the paths specified by this index tree is balanced somehow.

Generally, the Algorithm 1 in our previous work [33] constructs a tree that may be unbalanced, especially when sensor nodes are distributed unevenly in the network. Since [33] does not cache sensory data in leaf head nodes, leaf head nodes gather sensory data from sensor nodes and route these data to the SN. An unbalanced tree for a skewed distribution of sensor nodes prolongs the network lifetime, as evidenced by the experimental evaluation conducted in [33]. On the other hand, leaf head nodes require caching sensory data locally, as presented by Section 4 in this article, and sensory data, whose variation is beyond an allowed threshold, are not required to be routed to the SN. Therefore, when sensory data of most sensor nodes do not vary dramatically, the effort for routing sensory data to the SN should be lighter than that of [33] in the network, but the effort of leaf head nodes should be heavier due to the caching of sensory data locally. Intuitively, an unbalanced tree should a have much greater number of head nodes whose children include sensor nodes and head nodes, while all head nodes should be leaf head nodes for a half tree [38]. Therefore, more hops are to be involved when routing sensory data to the sink node in a hop-by-hop manner, especially when the path is longer in hops. To facilitate the caching mechanism upon leaf head nodes as presented in Section 4, a relatively balanced tree is constructed for organizing sensor nodes in this article.


**Algorithm 1** Index tree construction.
**Require:**
*LN_set_*: the set of leaf nodes with inverted files**Ensure**: *rt*: the root of the index tree1:*TN_set_* ← set of leaf nodes and initially set to *LN_set_*2:*nTN_set_* ← set of tree nodes recording newly-merged nodes3:**while** |*TN_set_*| > 1 **do**4: *wgt*(*nd*_1_, *nd*_2_) ← *calNgbNdWgt*(*nd*_1_, *nd*_2_) as presented by Algorithm 2 in our previous work [33], where *nd*_1_ and *nd*_2_ are neighboring nodes in *TN_set_*5: **while** ∃ *nd*_1_, *nd*_2_ ∈ *TN_set_*: *nd*_1_ and *nd*_2_ are neighboring nodes **and**
*wgt*(*nd*_1_, *nd*_2_) is the biggest **do**6:  *tn* ← merge tree nodes *nd*_1_ and *nd*_2_7:  *nd*_1_*, nd*_2_ ← children of *tn*8:  *tn*.*IvtF* ← *nd*_1_*.IvtF* ∪ *nd*_2_*.IvtF*9:  *TN_set_* ← *TN_set_* - { *nd*_1_, *nd*_2_ }10:  *nTN_set_* ← *nTN_set_* ∪ {*tn*}11: **end while**12: *TN_set_* ← *nTN_set_* ∪ *TN_set_*13: *nTN_set_* ← ∅14:**end while**15:*rt* ← *tn*


**Algorithm 2** Data synchronization.
**Require:**
*SN_IN_*: a set of sensor nodes that are contained in an intermediate node (IN)**Ensure:** sensory data cached in IN are updated1:**for** each *v_sn_* ∈ *SN_IN_*
**do**2:_ _
$$v_{sn}.val_{vsn}^{df}\leftarrow |v_{sn}.val_{vsn}^{cur}-v_{sn}.val_{vsn}|$$3: **if**
$$v_{sn}.val_{vsn}^{df}>thrh_{atr}$$
**then**4:  
$$v_{sn}.{\text{val}}_{vsn}\leftarrow v_{sn}.val_{vsn}^{cur}$$5:  
$IN.vec_{IN}^{v_{sn}}.ts_{fmS}\leftarrow \text{current}\;\text{time}\;\text{slot}$6:  **if**
*v_sn_*.*sd_stt_* is active **then**7:   
$IN.vec_{IN}^{v_{sn}}.val_{vsn}\leftarrow v_{sn}.val_{sn}^{cur}$8:  **else**9:   
$IN.vec_{IN}^{v_{sn}}.val_{vsn}\leftarrow null$10:  **end if**11: **end if**12:**end for**

**Figure 3:**
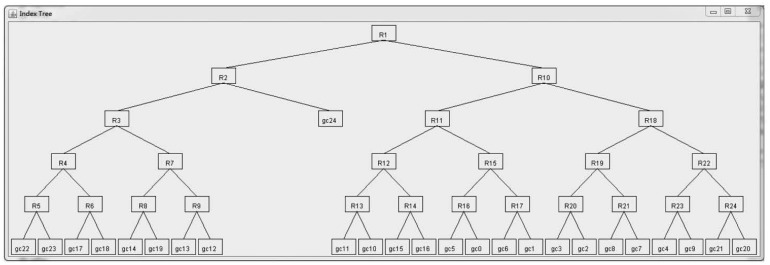
An example of the index tree constructed through Algorithm 1, where 1000 sensor nodes are deployed in the network region, and the skewness degree is set to 60%. The leaf nodes in the index tree correspond to the grid cells (e.g., *gc*_24_), and non-leaf nodes (e.g., *R*_5_) correspond to sub-regions containing multiple grid cells (e.g., *gc*_22_ and *gc*_23_).

As previously mentioned, the index tree to be constructed in this article should be a relatively balanced tree for a skewed distribution, rather than an unbalanced tree, as developed in our previous work [33]. The difference lies mainly in the tree node merging strategy As presented in Algorithm 1, after dividing the network region into square grid cells whose side-length is $\sqrt{2r}$ , we firstly get the set of leaf nodes (denoted *TN_set_*) with inverted files as presented by Algorithm 1 (Lines 1–13) in our previous work [33] (Line 1). Note that these leaf nodes corresponds to grid cells, rather than sensor nodes in the network. The weight (denoted *wgt*(*nd*1, *nd*2)) between neighboring nodes (denoted *nd*_1_ and *nd*_2_), which reflects the energy consumption when routing sensory data from one node to another, is calculated using the function *calNgbNdWgt*(*nd*_1_, *nd*_2_). As presented by Algorithm 2 in our previous work [33] (Line 4), this function returns the sum of weights among all pairs of neighboring grid cells in the corresponding neighboring sub-regions. If there are neighboring nodes (*i.e.*, *nd*_1_ and *nd*_2_) in *TN_set_* that can be merged, in other words, *wgt*(*nd*1, *nd*2) is the biggest (Line 5), *nd*_1_ and *nd*_2_ are merged as a newly-generated node *tn* (Lines 6–7), and the inverted files (denoted *tn*.*IvtF*, for instance) are processed accordingly (Line 8). Note that *tn* corresponds to a sub-region in the network, which is the merger of sub-regions for *nd*_1_ and *nd*_2_. After this merging procedure, *nd*_1_ and *nd*_2_ are removed from *TN_set_* (Line 9), while *tn* is inserted into *nTN_set_* as a candidate for the next merging procedure (Line 10). This procedure iterates until all neighboring nodes in *TN_set_* have been examined and processed. Thereafter, *TN_set_* and *nTN_set_* are updated accordingly (Lines 12–13). The symbol ∅ in Line 13 means an empty set. This tree construction procedure terminates one there is only one node left in *TN_set_* (Line 3), which corresponds to the root node of the index tree (Line 15). An example of the index tree constructed through this algorithm is shown in Figure 3, which is a binary tree and more balanced than the tree as shown in Figure 5 in our previous work [33]. Specifically, 25 grid cells, as shown in Figure 2, correspond to the leaf nodes, and sub-regions composed of multiple grid cells correspond to non-leaf nodes, in the index tree. This index tree construction is to facilitate the popularity-based cooperative caching mechanism to be presented in the following sections. The time complexity of Algorithm 1 is *O*(*n* × log_2_ *n*), where *n* is the number of leaf nodes in, while log_2_ *n* is the height of the index tree.

Generally, a node in an index tree corresponds to a sub-region containing a subset of tree nodes and/or sensor nodes, and these tree nodes are responsible for query propagation and sensory data routing to the *SN*. The low-energy adaptive clustering hierarchy protocol (*LEACH*) [39] is adopted for the head node selection in these sub-regions or grid cells, where these sub-regions or grid cells correspond to the clusters as presented by Heinzelman *et al.* [39]. LEACH incorporates randomized rotation of high-energy sensor nodes as the head nodes to avoid draining the energy of head nodes. Consequently, the energy consumption of being a cluster head node is distributed and balanced among sensor nodes. This strategy facilitates the prolongation of the network lifetime to some extent. Optimal head nodes should be located at the center of a cluster [40,41]. Therefore, a higher priority is given to sensor nodes that are closer to the center of sub-regions or grid cells when voting for head nodes.

### 3.2. Two-Tier Cooperative Caching Model

Applications leveraging WSNs may require gathering sensory data in a short latency, while minimizing the energy consumption of the network. Queries issued periodically and continuously for gathering sensory data of multiple attributes should induce high communication cost, which may be above the network capability. Without loss of generality, we divide the time duration into time slots as our previous work [42], and queries are assumed to be conducted in batches in each time slot. Note that sensory data in some applications, such as health or wildlife monitoring, may not change dramatically within a certain time duration. Besides, some applications may tolerate a bias of sensory data, when the variation of these data can satisfy a certain constraint. These suggest that sensory data may be valid for the applications within some time slots after the sensing time point. Therefore, these sensory data may be appropriate to reuse for answering the forthcoming queries, rather than fetching from the network in a real-time fashion [42]. To facilitate this sensory data reusability strategy, this article proposes a cooperative data caching mechanism, where sensory data are cached in the memory of: (i) the SN; and (ii) head nodes of grid cells, which correspond to the leaf nodes in the index tree. We use the notion of intermediate nodes (INs) to represent these leaf head nodes in the following. Generally, the SN, which has a larger memory space and more capability in computation than sensor nodes, is responsible for caching the bulk (may be not all) of sensory data from the network. An IN is required to cache sensory data of sensor nodes in the corresponding grid cell. Since there are usually a limited number of sensor nodes in each grid cell, an IN is assumed to have enough memory space and computational capability for processing sensory data of all sensor nodes in the corresponding grid cell. To facilitate the query processing mechanism in the following sections, we define a wireless sensor network as follows:

**Definition 1** (WSN). *A wireless sensor network is a tuple wsn* = (*SN*, *V_IN_*, *V_sn_*, *ATR*), *where*:

- SN is the sink node of this network.
- V_IN_ is a set of intermediate nodes, which are responsible for handling sensory data in grid cells.
- V_sn_ is a set of sensor nodes in the network.
- ATR is a set of attributes to be sensed by V_sn_.

Given a sensor node *v_sn_* ∈ *V_sn_*, *v_sn_* is defined in terms of the vector:

(4) $$v_{sn}=<sd_{id},sd_{atr},sd_{stt},val_{vsn}>$$

where *sd_id_* means the identifier of this sensor node *v_sn_*, *sd_atr_* ∈ *ATR* represents the single attribute to be sensed by *v_sn_*, *sd_stt_* specifies the status of *v_sn_*, which can be active or inactive, and *val_vsn_* is the sensory data that may be cached in the corresponding intermediate node *v_IN_* ∈ *V_IN_* and the SN. Note that *val_vsn_* is not the sensory data $val_{vsn}^{cur}$ at this moment, and *val_vsn_* is to be replaced by $val_{vsn}^{cur}$ only when the bias between *val_vsn_* and $val_{vsn}^{cur}$ is above an allowed threshold *thrd.* In this case, *v_sn_* should:

- report $val_{vsn}^{cur}$ to the corresponding *v_IN_* for the update of this sensory data in *v_IN_* when *v_sn_* is in the status of active, which means that *v_IN_* is interested in queries in recent time slots. Note that the SN should synchronize with *v_IN_* for retrieving the last sensory data when a query interest a grid cell *v_IN_.* When *v_IN_* is not interested in queries for a certain number of recent time slots, *v_IN_* is assumed not interested currently, and all sensor nodes in *v_IN_* are set to the status of inactive. On the other hand, when *v_IN_* is interested in a query, all sensor nodes in *v_IN_* are reset to the status of active immediately.
- Otherwise, *v_sn_* should report a sensory data change notification message to *v_IN_* when *v_sn_* is in the status of inactive, and thereafter, *v_IN_* will invalidate the sensory data in the cache.

Note that *thrd* is determined according to: (i) the kind of attribute to be sensed by *v_sn_;* and (ii) the specific requirement of certain applications. As for the sensory data synchronization procedure, we refer to Section 4.1 for the details.

An intermediate node *v_IN_* should cache sensory data of all sensor nodes in the corresponding grid cell, in terms of the vector for a sensor node *v_sn_:*

(5) $$vec_{IN}^{vsn}=<sd_{id},sd_{atr},sd_{stt},val_{vsn},ts_{fmS},ts_{toSN}>$$

where *sd_id_, sd_atr_, sd_stt_* and *val_vsn_* are the same as those of *v_sn_*, respectively. *ts_fms_* records the time slot when *val_vsn_* is reported from the sensor node, while *ts_toSN_* records the time slot when *val_vsn_* is retrieved by SN. When *ts_fms_* is the same as *ts_tosN_*, the last sensory datum of *v_sn_* has been cached in the SN if the SN has enough memory space. Otherwise, the cached sensory data in the SN is not synchronized with that in the corresponding IN. Note that $vec_{IN}^{vsn}.val_{vsn}$ should be set to null when *v_sn_* reports a sensory data change notification message to the IN.

The SN caches sensory data of sensor nodes in terms of the vector:

(6) $$vec_{SN}^{vsn}=<sd_{id},gc_{id},sd_{atr},val_{vsn}>$$

where *sd_id_*, *sd_atr_* and *val_vsn_* are the same as those of sensor node *v_sn_*, respectively. *gc_id_* refers to the ID of the corresponding grid cell where *v_sn_* lies. Since the SN has a limited storage capability, sensory data of sensor nodes, which are the most popular according to the recent query history, are to be cached in the SN. The popularity computation of grid cells is presented in Section 4.2.

## 4. Query Processing with Cache Mechanism

Given a network deployed in a certain region where multiple kinds of attributes are interested, queries are to be issued periodically and continuously according to the requirement of certain applications. Generally, a multiple-attribute query can be described in terms of a three-dimensional vector, including: (i) a query region; (ii) a set of interested attributes; and (iii) a certain time slot. A query region is typically represented by a rectangle. As mentioned in Section 3.1, the network region is divided into grid cells. Consequently, a query region is transferred to a set of grid cells with the minimum number, which can cover the rectangle prescribed by this query. Formally, a query is defined as follows:

**Definition 2** (Query). *A query is a tuple q* = (*tm*, *qr*, *ATR*)*, where:*

- tm is the time slot when the query q is issued.
- qr is the query region, which is represented by a set of corresponding square grid cells.
- ATR is a set of attributes interested in q.

Given a query *q* = <*tm*, *qr*, *ATR_q_*> where *q*.*qr* is rewritten as a set of grid cells *GC_q_* (*i.e., q*.*qr* = *GC_q_*), *q* returns sensory data at *q*.*tm* of sensor nodes: ∀ *v_sn_* ∈ *GC_q_*: (*v_sn_*.*sd_id_* is contained by a grid cell within *GC_q_*) ∧ (*v_sn_*.*sd_atr_* ∈ *ATR_q_*).

It is worth mentioning that in certain domain applications, including health monitoring, queries are typically issued continuously and periodically. The points of interest should be within certain sub-regions for certain contiguous time slots, while evolving moderately to neighboring sub-regions [15]. In this context, when the sub-regions for the queries in contiguous time slots have overlapping sub-regions, query results in the previous time slots should be valid and (partially) reusable for answering the forthcoming queries. On the other hand, queries are answered independently by the SN. Generally, for a certain query *q* to be processed by the SN, *q* is answered by a cooperative caching mechanism, including the following steps:

- Step 1: The SN determines a set of grid cells *GC_q_* of a minimum number that can cover the query sub-region *q*.*qr*.
- Step 2: The SN synchronizes with the intermediate nodes (INs), which are actually the head nodes of *gc_q_* ∈ *GC_q_* in the index tree, for retrieving the last sensory data. It is worth mentioning that the SN may cache sensory data of some, but not all, INs. Intuitively, a flag is adopted for specifying whether sensory data of a certain IN has been cached in the memory space of SN or not.
- Step 3: An IN examines the freshness of cached sensory data for each sensor node (denoted *v_sn_*) in the corresponding grid cell *gc_q_.*
  – When *v_sn_*.*ts_fmS_* = *v_sn_*.*ts_toSN_*, which suggests that sensory data cached in IN is up-to-date, and is synchronized with that cached in the SN, a flag indicating this scenario is sent to the SN, whereas sensory data of *v_sn_* do not need to be forwarded to the SN.
  – Otherwise, sensory data cached in the IN are not up-to-date. Consequently, the head node of the IN requests to retrieve the last sensory datum from *v_sn_*, which is to be routed to the SN afterwards.
  Note that the status of *v_sn_* is set to active when *v_sn_* is interested in queries currently and/or in recent time slots. The reader is referred to Section 4.1 for the details of the sensory data synchronization mechanism between an IN and the sensor nodes contained therein.
- Step 4: When the SN gets sensory data of all sensor nodes in each grid cell *gc_q_* ∈ *GC_q_*, the answer to the query *q* is aggregated. Sensory data, which were cached in the memory space of the SN and are not consistent with the last ones retrieved from INs, are updated accordingly. Note that the SN usually has a constraint in storage and computational capabilities. As presented in Section 4.2, when sensory data of some grid cells are retrieved from INs, sensory data cached in the SN, which may not be reused for answering the forthcoming queries, should be removed, and the released storage capability can be adopted for caching more popular sensory data.

As discussed at Steps 2–4, the technical details of our two-tier cooperative caching mechanism on the SN and IN for query answering are presented in Section 4.3.

### 4.1. Sensory Data Synchronization for INs and Corresponding Sensor Nodes

As discussed, in certain applications of WSNs, the points of interest may be within certain regions in certain time durations, while evolving to neighboring sub-regions moderately and continuously. This suggests that a certain grid cell may be interested in applications of some time durations, while not in the others. To reduce the energy consumption, sensor nodes, which do not contribute to answering the queries in a few recent time slots, are set to the status of inactive. Therefore, these sensor nodes are not required to send sensory data to the IN at each time slot afterwards. Instead, when their sensory data have been changed dramatically and the variation is not tolerable for applications, a flag indicating this situation is sent to the IN. It is worth mentioning that the strategy of active and inactive is different from adaptive sleeping [43,44] and duty-cycle [45,46] strategies, where sensor nodes do not sense environmental variables and respond to queries. For simplicity, we assume that sensor nodes decide to send either sensory data or a flag (data of a bit usually) to the IN according to the status of active or inactive. This strategy decreases the amount of data to be sent and thus reduces the energy consumption. In fact, adaptive sleeping and duty-cycle strategies complement our technique, and when applied, energy consumption should be further reduced.

The sensory data synchronization strategy for the IN and contained sensor nodes is presented in Algorithm 2. For each sensor node *v_sn_* in each IN, *v_sn_* examines whether the sensory data at this moment (denoted $v_{sn}.val_{vsn}^{cur}$ have been changed dramatically with respect to the sensory data reported to the IN (denoted *v_sn_*.*val_vsn_*) in previous time slots (Line 2). If the variation $v_{sn}.val_{vsn}^{df}$ , which is represented by the absolute value of the difference between $v_{sn}.val_{vsn}^{cur}$ and *v_sn_*.*val_vsn_*, is above an allowed and pre-specified threshold (denoted *thrh_atr_*), *v_sn_* should report this data change situation to the IN (Line 3). In this case, *v_sn_* sets its reported sensory data *v_sn_*.*val_vsn_* as the current value $v_{sn}.val_{vsn}^{cur}$ (Line 4). $IN.vec_{IN}^{vsn}.ts_{fmS}$ is set to the current time slot (Line 5). $v_{sn}.val_{vsn}^{cur}$ is reported to the IN for updating the cached data when *v_sn_* is in the status of active. To be specific, $IN.vec_{IN}^{vsn}.val_{vsn}$ is replaced by $v_{sn}.val_{vsn}^{cur}$ (Lines 6–7). Otherwise, a flag indicating this dramatic data change situation is reported to the IN, and the corresponding sensory data for *v_sn_* cached in IN is set to null (Lines 8–9). Consequently, sensory data cached in the IN are synchronized with those of sensor nodes contained in this IN. Note that this data synchronization procedure is performed at each time slot, which facilitates the answering of queries as presented in Section 4.3.

The time complexity of Algorithm 2 is *O*(*m_gc_*), where *m_gc_* is the maximum number of sensor nodes contained in a grid cell. The worst case occurs when sensory data of all sensor nodes have been changed dramatically, and thus, all sensory data cached in the IN have to be updated consequently.

### 4.2. Popularity-Based Sensory Data Replacement Mechanism in the SN

As mentioned in Section 3.2, sensory data of certain sensor nodes may not change dramatically in certain time durations, and domain applications may work well when the bias of sensory data is beyond a certain threshold with respect to the data in real time. In this setting, sensory data cached in the memory space of the SN may be appropriate to be (partially) reused for answering the forthcoming queries, rather than retrieving from the network in real time. This mechanism should reduce the effort of sensory data sensing, gathering and routing and, thus, should prolong the network lifetime to some extent. Note that the SN usually has limited storage and computational capabilities, which may not be capable of caching sensory data of all sensor nodes in the network. Therefore, sensory data that have a high possibility of being reused for answering the forthcoming queries should be cached. Generally, queries are issued at each time slot. When fresh sensory data of sensor nodes are retrieved from the network, these fresh sensory data should be used for updating the obsolete counterparts if cached in the SN previously. On the other hand, sensory data cached in the SN, which may not contribute to the forthcoming queries, should be removed, and the released memory space is used for caching the fresh sensory data. This section proposes a mechanism for sensory data replacement in the cache of the SN depending on the popularity of sensory data. Note that the network is divided into square grid cells in this article, and a grid cell (denoted *gd*), with an interested attribute (denoted *atr*), is considered as an atomic unit for query processing. Intuitively, the vector *vc* = <*gd*, *atr* > is applied to represent the index of the set of sensory data to be cached in the SN.

Given a set of sensory data represented by the vector *vc_i_* = <*gc_i_*, *atr_i_*>, where these data are (i) cached in the SN or (ii) were not cached in the SN and are retrieved from the network at this moment, we calculate the popularity $pop_{\upsilon c}^{i}$ of *vc_i_* according to (i) the history of queries conducted in the previous *k* time slots and (ii) the size of sensory data contained by *vc_i_*. A large value of $pop_{vc}^{i}$ means that *vc_i_* is higher in possibility of being cached in the SN and of being interested in the queries in the consequent time slots. Specifically, $pop_{vc}^{i}$ is calculated using the following formula:

(7) $$pop_{vc}^{i}=\frac{1}{sz_{vc_{i}}}\times \sum_{j=1}^{k}\left(\alpha^{j}\times \frac{f_{i}^{j}}{f_{all}^{j}}\right)$$

where *sz_vci_* means the size of sensory data in *vc_i_*, which is proportional to the number of sensor nodes contained in *gc*_i_ accompanying the attribute *atr_i_* and the size of sensory data for the attribute *atr_i_*. This indicates that the larger the size of *vc_i_* is (*i.e.*, $\frac{1}{sz_{vc_{i}}}$ is smaller), the higher the possibility that *vc_i_* is removed from the SN. $f_{i}^{j}$ represents the number of queries that are interested in *vc_i_* at the time slot *j*, while *f_all_* represents the number of all vectors *vc* = <*gd*, *atr*> interested in all queries issued at the time slot *j*. Generally, the more frequently that the *vc_i_* is interested in queries, the larger the popularity $pop_{vc}^{i}$ is. The parameter *α* corresponds to an attenuation coefficient, which is set to a value between zero and one. In fact, *α* reflects the importance of queries conducted in a recent, while different, time slot. Intuitively, the more recent queries the *vc_i_* is interested in, the larger the popularity $pop_{vc}^{i}$ is.

Given a set of *VC* = {*vc*_1_, *vc*_2_, ⋯} that are gathered at a certain time slot, the popularity $pop_{vc}^{i}$ of *vc_i_* ∈ *VC* is calculated using Formula 7. $pop_{vc}^{i}$ are ranked according to their values. Sensory data of *vc_i_* are cached in the SN until not enough storage capability in the SN is available for the next. These cached sensory data are used for facilitating the cooperative caching mechanism, as detailed in the following.

### 4.3. Query Processing with Two-Tier Cooperative Caching Mechanism

Leveraging sensory data cached in the memory space of INs and the SN, we propose a two-tier cooperative caching mechanism for answering periodic queries. As presented in our previous work [33], the network region is divided into square grid cells. A two-dimensional matrix is used to represent grid cells, where each grid cell is accompanied by an identifier (denoted *gc_id_*). *gc_id_* is computed based on the value of (i) row (denoted *row*) and column (denoted *col*) coordinates of the grid cell and (ii) the columns of the grid cell in the matrix (denoted *cols*). Specifically, *gc_id_* = *row* × *cols* + *col*. An example is shown in Figure 2, where *pi* (*i* = 1, 2, …) represents sensor nodes sensing various kinds of attributes. As for the grid cell containing sensor node *p*17, its grid cell ID is computed as 7 = 1 × 5 + 2, and this grid cell is denoted as *gc*_7_. The first grid cell, which contains *p*1, as shown in Figure 2, is denoted as *gc*_0_.

Given a query *q* to be answered, *q* is rewritten for determining a minimum set of grid cells that *q*.*qr* covers. When a grid cell is intersected with *q*.*qr*, this grid cell is counted. An example is shown in Figure 2, where the query region is represented using a rectangle with dotted lines, and grid cells for *q*.*qr* are marked in gray. The attributes that *q*.*ATR* includes are represented using a bitmap. An example is shown in Table 2, where one means that the query interests in the corresponding attribute, while zero means it is not. The parameter k represents the number of attributes to be sensed in a certain network.

When answering a query *q*, sensory data of multiple attributes in a certain grid cell should be gathered and aggregated once. An example is the attributes *atr*_1_ and *atr*_3_ with respect to grid cells *gc*_0_, *gc*_1_, *gc*_2_, *gc*_5_, *gc*_6_ and *gc*_7_, as shown in Figure 2 and Table 2. To facilitate the query processing, a GC-attribute table is adopted to clearly represent this relation for grid cells and interested attributes. For instance, Table 3 shows the relation for grid cells (*i.e.*, *gc*_0_, *gc*_1_, *gc*_2_, *gc*_5_, *gc*_6_ and *gc*_7_) and interested attributes (*i.e., atr*_1_ and *atr*_3_)*.* It is worth mentioning that a grid cell with a single attribute of interest is considered as the atomic unit for query processing and the sensory data caching mechanism as presented in Section 4.2. Generally, Table 3 is an example for the relation of grid cells and attributes for a single query, where one means that the corresponding attribute in the certain grid cell is interested in the query, whereas zero means it is not. A table in this format can also be applied to specify the relation of grid cells and attributes aggregated for multiple queries that are to be processed concurrently at a certain time slot. For simplicity, as presented by Algorithm 3, a single query is considered for the query processing procedure leveraging the cooperative caching mechanism, whereas multiple queries can be handled in a similar fashion. Besides, a table, which is named the GC-SNCache table and an example shown in Table 4, is used to represent sensory data of grid cells that are cached in the SN currently, where one means that the corresponding attribute in the certain grid cell has been cached in the memory space of the SN, whereas zero means it is not. Note that the SN is usually limited in storage and computational capabilities and should cache sensory data of grid cells, which have a high possibility of being covered by the forthcoming queries.

As presented by Algorithm 3, when a query *q* is issued at a certain time slot, *q* is rewritten and a set of grid cells (denoted $GC_{set}^{q}$ are retrieved that covers *q*.*qr*, as illustrated by Figure 2 (Line 1). If the attributes interested in the query *q* (denoted *q*.*ATR*) are not sensed by the sensor nodes in the sub-region corresponding to the tree node *tn* or the query region (*i.e.*, $GC_{set}^{q}$ ) and the sub-region contained in the tree node (denoted *tn*.*GC*) have no overlapping areas (Line 2), no sensory data should be returned, and the tree node *tn* does not contribute to the query *q* (Line 3). The symbol ∅ in Line 2 specifies an empty set. Otherwise, *tn* contains sensor nodes that can contribute to the answering of the query *q*.

Generally, two scenarios are considered leveraging the fact of whether *tn* corresponds to a leaf node in the index tree or not. When *tn* does not reflect a leaf node in the index tree (Line 5), the left and right children of *tn* are processed independently, and their results are represented as $q_{rt}^{lf}$ and $q_{rt}^{rt}$ , respectively (Lines 6–7). The querying result of *q* (denoted *q_rt_*) is assembled as the aggregation of $q_{rt}^{lf}$ and $q_{rt}^{rt}$ (Line 8).

On the other hand, when *tn* reflects a leaf node in the index tree, *tn* corresponds to a grid cell actually. In this setting, the intermediate node (IN) with respect to *tn* is identified (Line 10), and the set of sensor nodes (denoted $V_{sn}^{all}$ ) contained in *tn*, which contribute to the answering of *q*, are retrieved (Line 11). The status of sensor nodes in $V_{sn}^{all}$ is changed to active, when it is inactive currently (Line 12).


**Algorithm 3** QueryProcCoopCaching
**Require:**
*q*: a query issued at a certain time slot*tn*: a node in the index tree**Ensure:**
*q_rt_*: the result for the query *q*1
$GC_{set}^{q}$ ← set of grid cells that are covered by *q*.*qr*2**if**
*q*.*ATR* ∩ *tn*.*IvtF* = ∅ **or**
$tn.GC\cap GC_{set}^{q}=\emptyset$
**then**3 **return**4**end if**5**if**
*tn* has children **then**6 
$q_{rt}^{lf}$ ← *QueryProcCoopCaching*(*q*, *tn*.*lfCld*) when the left child *tn*.*lfCld* exists7 
$q_{rt}^{rt}$ ← *QueryProcCoopCaching*(*q*, *tn*.*rtCld*) when the right child *tn*.*rtCld* exists8 *q_rt_* ← aggregation of 
$q_{rt}^{lf}$ and 
$q_{rt}^{rt}$9**else**10 IN ← get the intermediate node that contains *tn*11 
$V_{sn}^{all}$ ← sensor nodes in *tn*, where ∀ 
$v_{sn}\in V_{sn}^{all}$
**and**
*v_sn_*.*sd_str_* ∈ *q*.*ATR*12 *V_sn_.sd_stt_*← active, where 
$\forall \upsilon_{sn}\in V_{sn}^{all}$
**and**
*v_sn_*.*sd_stt_* = inactive13 **for each**
$v_{sn}\in V_{sn}^{all}$
**and** (*IN*.*v_sn_*.*ts_fmS_* ≠ *IN*.*v_sn_*.*ts_toSN_*
**or**
*IN*.*v_sn_*.*ts_toSN_* = *null*) **do**14   *V_sn_* ← *V_sn_* ∪{*v_sn_*}15 **end for**16 IN retrieves sensory data from grid cell *tn* for *V_sn_*, replaces cached sensory data in IN for *V_sn_* and sets *IN*.*v_sn_*.*ts_fms_* and *IN*.*v_sn_*.*ts_toSN_* to the current time slot for ∀ *v_sn_* ∈ *V_sn_*17 **if** grid cells for *tn* and *q*.*ATR* are not cached in the SN according to the GC-SNCache table **then**18  **for each**
$v_{sn}\in V_{sn}^{all}$19   *V_sn_* ← *V_sn_* ∪{*v_sn_*}20 **end for**21 mark all grid cells for *tn* and *q*.*ATR* as being cached in the GC-SNCache table22 **end if**23 **for each**
*v_sn_* ∈ *V_sn_*
**do**24  
$$SN.vec_{SN}^{vsn}.val_{vsn}\leftarrow IN.vec_{IN}^{vsn}.val_{vsn}$$, where 
$v_{sn}.sd_{id}=SN.vec_{SN}^{vsn}.sd_{id}=IN.vec_{IN}^{vsn}.sd_{id}$25 **end for**26 **for** ∀ 
$v_{sn}\in V_{sn}^{all}$
**and**
$v_{sn}.sd_{id}=SN.vec_{SN}^{vsn}.sd_{id}$
**do**27  
$q_{rt}\leftarrow \text{insert}SN.vec_{SN}^{vsn}.val_{vsn}$28 **end for**29**end if**

For each sensor node $v_{sn}\in V_{sn}^{all}$, when the sensory data for *v_sn_* cached at the corresponding IN are not aligned with those cached in the SN (indicated by *IN*.*v_sn_*.*ts_fmS_* ≠ *IN*.*v_sn_*.*ts_toSN_* or *IN*.*v_sn_*.*ts_toSN_* = *null*, where *IN*.*v_sn_.ts_fmS_* ≠ *IN*.*v_sn_*.*ts_toSN_* suggests that the last sensory data provided by *v_sn_* have not been synchronized with those cached in the SN, while *IN*.*v_sn_*.*ts_toSN_* = *null* suggests that the sensory data for *v_sn_* have never been reported to the SN) (Line 13), the last sensory datum for *v_sn_* should be retrieved from the network, and the time slots, which reflect the time when (i) sensor nodes report their sensory data to the IN (denoted *ts_fmS_*) and (ii) the IN reports sensory data of certain sensor nodes to the SN (denoted *ts_toSN_*), are updated accordingly (Line 16). It is worth mentioning that, when the status of *v_sn_* is inactive, the IN may not cache the sensory data for *v_sn_*, according to our data synchronization mechanism presented by Algorithm 2. As for the sensor nodes whose cached sensory data are consistent between the SN and the corresponding IN, according to the GC-SNCache table, these sensory data are not required to be retrieved from the network and to be routed to the SN. Instead, the sensory data cached in the SN are adopted for answering the query *q*. This strategy should reduce the energy consumption, which may be unnecessary somehow. Otherwise, the GC-SNCache table is updated for showing the sensory data consistency between the SN and the corresponding IN at this moment (Lines 17 and 21). Note that when the SN does not cache the sensory data for sensor nodes in $V_{sn}^{all}$ (Line 17), the sensory data of all sensor nodes in $V_{sn}^{all}$ should be forwarded to the SN for answering the query *q* (Lines 18–20). Consequently, sensory data of all sensor nodes in $V_{sn}^{all}$ are synchronized between the SN and the corresponding IN (Lines 23–25). The result of the query *q*, which is represented by *q_rt_*, is assembled leveraging the sensory data cached in the SN accordingly (Lines 26–28).

The time complexity of Algorithm 3 is *O*(*n*×*m*), where *n* is the number of tree nodes to be examined in the index tree leveraging the inverted file, and *m* is the maximum number of sensor nodes in grid cells. The worst case occurs when all sensor node in the whole network are to be traversed. In this setting, all sensor nodes are required to report their sensory data to the SN.

## 5. Implementation and Evaluation

A prototype has been implemented in a Java program, and experiments have been conducted for evaluating our technique. In the following, we introduce the environment settings and discuss the results of our experiments.

### 5.1. Environmental Settings

Experiments are designed for evaluating our technique, and the factors considered include various skewness distributions for the network setting and various cache sizes in the SN. In the network setting, 1000 sensor nodes are generated and distributed unevenly with skewness degrees varying from 20%–80%. Intuitively, a skewness degree (denoted *sd*) is computed using the formula: $sd=\frac{dn-sn}{N}$ , where *dn* and SN refer to the number of sensor nodes in dense and sparse sub-regions, respectively, while *N* is the number of sensor nodes deployed in the network (*i.e.*, *N* = *dn* + *sn*) [33]. For instance, assuming a network contains *N* (e.g., *N* = 1000) sensor nodes, the network region is divided into four sub-regions, which are rectangular in shape and the same in geographical size. A skewness degree is set to *sd* (e.g., *sd* = 60%). Consequently, *n* × (1 − *sd*) (1000 × (1 − 60%) = 400) sensor nodes are deployed evenly in the whole network, while the remaining *n* × *sd* (1000 × 60% = 600) sensor nodes are distributed to any two dense sub-regions in a random fashion. Therefore, a network region with *N* sensor nodes, whose distribution follows a skewness degree *sd*, is constructed.

Experiments are performed on a desktop with an Intel(R) Core(TM) i5-2400 CPU at 3.10 GHz, a 4-GB memory and a 32-bit Windows system. Parameter settings for our experiments are presented in Table 5. Note that several, but typically not too many, kinds of attributes are interested in domain applications. Without loss of generality, 10 kinds of attributes are assumed interested in the experiments. A sensor node is randomly assigned an attribute to be sensed, and each kind of attribute is sensed by around 1000/10 = 100 sensor nodes. The network region is set to 350 m × 350 m, which is divided into square grid cells with the same geographical size. The side-length of grid cells is set to $\sqrt{2r}$ [40], where *r* = 50 m is the communication radius of sensor nodes. The size of cache in the SN is set to a number between 500 and 900, which means that the SN can accommodate sensory data for 50–900 sensor nodes, respectively. The attenuation coefficient *α* and the number of preceding time slots *k* used in Equation (7) are set to 0.6 and four, respectively. Queries are issued every 2 min, and sensory data between INs and the corresponding sensor nodes are synchronized every 5 min, when the status of these sensor nodes is inactive. These parameters can be set to other values if appropriate.

### 5.2. Experimental Evaluation

An index tree is constructed through recursively merging neighboring sub-regions when forwarding messages of the same size in between is minimum in energy consumption. An example is shown in Figure 3, where the skewness degree is set to 60%. Leaf nodes are denoted by the IDs of correspond grid cells, as mentioned in Section 3.2, and *R_i_* (*i* = 1, 2, …) represents non-leaf nodes, which are (sub-)regions composed of several neighboring grid cells. For instance, *R*_5_ represents a sub-region whose grid cells are *gc*_22_ and *gc*_23_, while *R*_2_ is composed of *R*_3_ and grid cell *gc*_24_. Query answering is performed leveraging this index tree for data gathering, aggregation and routing to the SN.

Our technique is evaluated with respect to four types of queries [33] leveraging the sub-region and attributes of interest, and a query is denoted as *q* = <*tm*, *qr*, *ATR_q_*>:

- Single-attribute query in the whole region (SAQWR): *q* retrieves sensory data of all sensor nodes in the whole network region (*i.e.*, *q*.*qr* = *nr*) for a certain attribute (*i.e.*, |*q*.*ATR*| = 1). *nr* specifies the whole network region. Sensory data of the attribute *q*.*ATR* at *nr* are gathered and routed to the SN at a certain time slot *q*.*tm.*
- Single-attribute query in a sub-region (SAQSR): The difference between SAQSR and SAQWR lies in the query region. SAQSR retrieves sensory data of sensor nodes in a sub-region of the network (*i.e., q*.*qr* ⫋ *nr*) with a certain attribute *q*.*ATR* (*i.e., |q*.*ATR|* = 1).
- Multi-attribute query in the whole region (MAQWR): *q*.*qr* is the whole network region (*i.e.*, *q*.*qr* = *nr*)*. q*.*ATR* contains some, but not all, attributes. Generally, sensory data of the attributes in *q*.*ATR* at *nr* are gathered and routed to the SN at *q*.*tm.*
- Multi-attribute query in a sub-region (MAQSR): The difference between MAQWR and MAQSR lies in the query region where MAQSR retrieves sensory data of sensor nodes in a sub-region of the network (*i.e.*, *q*.*qr* ⫋ *nr*) with multiple attributes.

**Figure 4:**
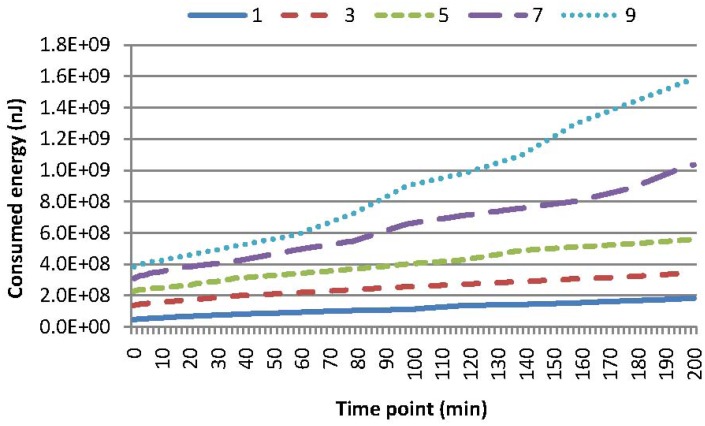
Comparison of the accumulated energy consumption for multi-attribute query in the whole region (MAQWR), where the number of attributes are set to 1, 3, 5, 7 and 9, respectively. The gradient of the curves represents the ratio of energy consumption for query answering. This figure shows that when the cache size in the SN is relatively small and is not capable of caching all sensory data requested by a certain query, more sensory data should be replaced in the SN according to our data replacement mechanism, as presented in Section 4.2, and more energy is required for answering the query. For instance, in comparison with the case when the number of attributes is three, 198% (or 355%) more energy is consumed for the case when the number of attributes is seven (or nine).

**Figure 5:**
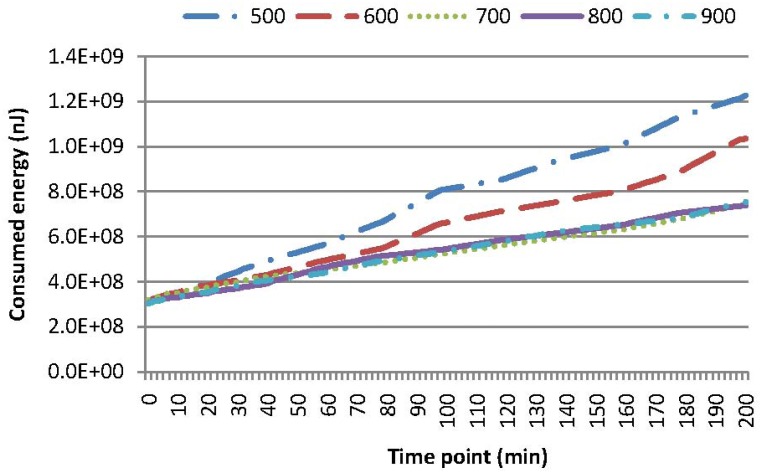
Comparison of the accumulated energy consumption for MAQWR, where the cache size of the SN is set to 500, 600, 700, 800 and 900, respectively. The gradient of the curves represents the ratio of energy consumption for answering the query. This figure shows that when the cache size of the SN is large enough to cache all sensory data requested by the query, the energy consumption is relatively small and steady. Otherwise, the energy consumption is much more and increases significantly. For instance, in comparison with the case when the cache size is set to 800, 66% (or 40%) more energy is consumed for the case when the cache size is set to 500 (or 600).

**Figure 6:**
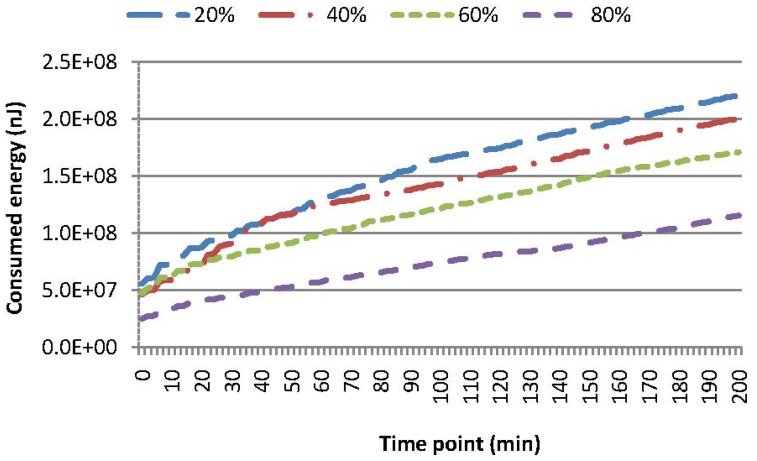
Comparison of the accumulated energy consumption for single-attribute query in the whole region (SAQWR), where the skewness degree for the sensor node distribution in the network is set to 20%, 40%, 60% and 80%, respectively. The gradient of the curves represents the ratio of energy consumption for answering the query. This figure shows that the bigger the skewness degree is, the less energy is consumed for answering the query. For instance, in comparison with the case when the skewness degree is set to 80%, 73% (or 91%) more energy is consumed for the case when the skewness degree is set to 40% (or 20%).

**Figure 7:**
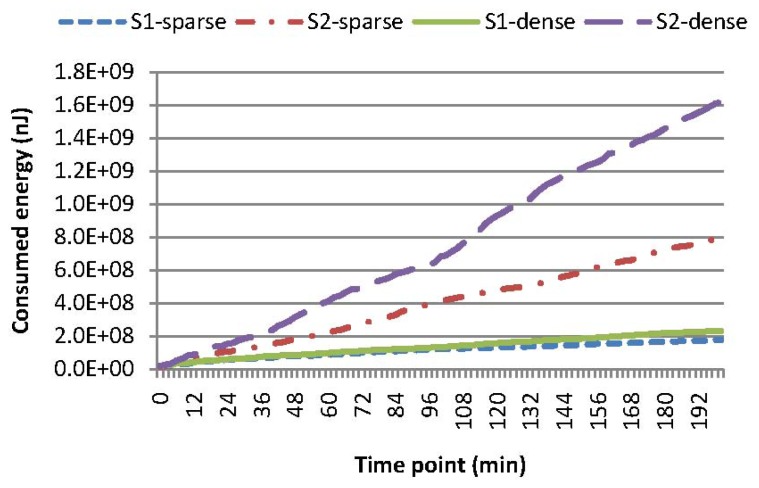
Comparison of the accumulated energy consumption for single-attribute query in a sub-region (SAQSR) with various query configurations, where *S1-sparse* means the sparse sub-regions with our cache mechanism, *S2-sparse* means sparse sub-regions without our cache mechanism, *S1-dense* means the dense sub-regions with our cache mechanism and *S2-dense* means the dense sub-regions without our cache mechanism. The gradient of the curves represents the ratio of energy consumption for answering the query. This figure shows that the energy consumption is decreased dramatically when our cooperative caching mechanism is adopted, especially when the sensors nodes are densely deployed in the network. For instance, 348% (or 599%) more energy is consumed for the case of *S2*-*sparse* (or *S2*-*dense*) than the case of *S1*-*sparse* (or *S1*-*dense*).

Experiments are conducted to evaluate the performance of these four kinds of queries leveraging our two-tier cooperative caching mechanism. Figure 4 compares the energy consumption of MAQWR, where the number of attributes varies as 1, 3, 5, 7 and 9, respectively. The cache size of the SN is set to 600, and the skewness degree is set to 60%. It is worth mentioning that Figure 4 shows the energy consumed in total from scratch, rather than that at a certain time point. The gradient of a curve corresponds to the energy consumed at a certain time point. The same principle holds for the energy consumption shown in Figures 5, 6–7. Intuitively, more energy is consumed when more attributes are of interest, since more sensor nodes are involved in sensory data gathering and aggregation. The energy consumption for the scenarios, where the number of attributes is 1, 3 or 5, is relatively small and stable. In contrast, the energy consumption for the scenarios, where the number of attributes is seven or nine, increases to a certain extent. Note that sensor nodes involved in the query answering should be 700 (or 900), when the number of interested attributes is seven (or nine). Since the cache size is 600, the sensory data of around 100 (or 300) sensor nodes cannot be cached in the SN. Hence, some sensory data have to be gathered from the network at each time slot, and the sensory data replacement mechanism is always enacted to cache sensory data with the highest popularity. These are the main causes for the increase of energy consumption. For instance, in comparison with the case when the number of attributes is three, 198% (or 355%) more energy is consumed for the case when the number of attributes is seven (or nine). Generally, the larger the cache size of the SN, the less the energy consumption in the network is. Caching in the SN should reduce the energy consumption to a certain extent, especially when the query region is relatively large and the number of interested attributes is relatively big.

**Figure 8:**
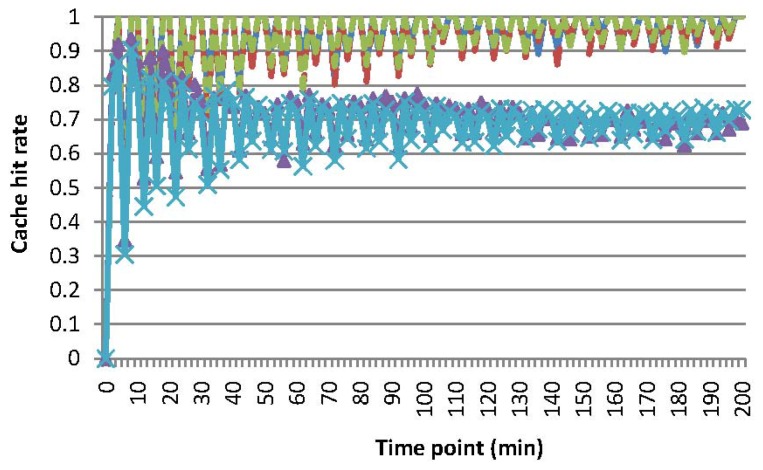
Comparison of cache hit rates for MAQWR, where the number of attributes are set to 1, 3, 5, 7 and 9, respectively. Similar to Figure 4, when the cache size of the SN is relatively small and is not capable of caching all sensory data requested by a certain query, the cache hit rates for sensory data cached in the SN decrease significantly (roughly from 95% down to 70%).

Figure 8 shows cache hit rates *hrt_cah_* for MAQWR. *hrt_cah_* is calculated as the ratio of $\left|SD_{cah}^{q}|/|{\mathrm{SD}}^{q}\right|$ , where (i) $SD_{cah}^{q}$ is the set of sensory data cached in the SN that contributes to answering of the query *q* and (ii) *SD^q^* is the set of sensory data of *q* inquiries. Without loss of generality, the value of sensory data is assumed to vary according to the formula: *val_vsn_* = log (k × *t_cur_* + 1) + C, where: (i) *t_cur_* is the current time; and (ii) k and C are constants, which are initially set to random values, and vary according to a normal distribution. Therefore, sensory data of sensor nodes are mostly different and change moderately. Figure 8 shows that cache hit rates for the scenarios, where the number of attributes is 1, 3 or 5, are quite high (roughly 95%), since the SN can have enough storage capability to cache almost all sensory data gathered in recent time slots. As for the scenarios where the number of attributes is seven or nine, cache hit rates are relatively lower (roughly 70%). Similar to the situation for energy consumption, a certain amount of sensory data has to be gathered from the network for query answering in real time. In addition, sensory data, which may be reused for answering the forthcoming queries, have to be removed from the cache by the data replacement mechanism, due to the limitation of the storage capability of the SN. Figure 8 shows that cache hit rates drop every 5 min. Since INs synchronize with sensor nodes in the corresponding grid cells every 5 min, sensory data, which have been changed remarkably, are retrieved from the network. These variations are routed to the SN, but these sensory data are not counted in $SD_{cah}^{q}$ , which induces the dropping of *hrt_cah_* consequently.

**Figure 9:**
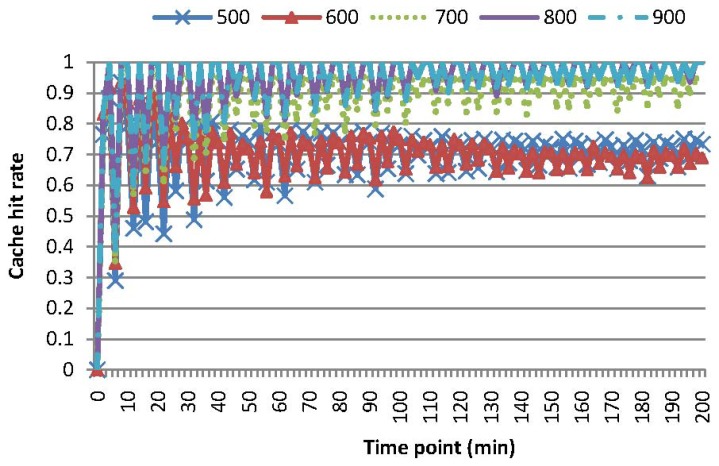
Comparison of cache hit rates for MAQWR, where the cache size of the SN is set to 500, 600, 700, 800 and 900, respectively. Similar to Figure 8, when the cache size of the SN is relatively small and is not capable of caching all sensory data requested by a certain query, the cache hit rates for sensory data cached in the SN decrease significantly (roughly from 95% down to 70%).

Figures 5 and 9 show the energy consumption and cache hit rates for MAQWR, where: (i) the cache size of the SN is set to 500, 600, 700, 800 and 900, respectively; and (ii) the number of attributes interested in queries is seven. Sensory data of around 700 sensor nodes are able to be cached in the SN. When the cache size is more than 700, much less energy (roughly 40%∼66% of a decrease) is consumed, as shown in Figure 5, and cache hit rates are much higher (roughly 25% of an increase) as shown in Figure 9. As discussed, the sensory data replacement mechanism and real-time data gathering from the network are the main causes of more energy consumption and cache hit rates dropping.

Figure 6 shows the energy consumption for SAQWR, where: (i) the skewness degree is set to 20%, 40%, 60% and 80%, respectively; and (ii) the cache size of the SN is set to 600. Note that around 100 sensor nodes are responsible for the query answering, and the cache of the SN can accommodate all of these sensory data. This figure shows that the bigger the skewness degree, the less the energy consumption is for the query answering in the whole network. For instance, in comparison with the case when the skewness degree is set to 80%, 73% (or 91%) more energy is consumed for the case when the skewness degree is set to 40% (or 20%). As presented in Section 3.1, our index tree is constructed through merging two neighboring sub-regions, where the energy consumption of forwarding the same size of messages is the least. Besides, our index tree is a relatively balanced tree, which reduces the path length of dense sub-regions for routing sensory data to the SN. Generally, our technique is more efficient, when sensor nodes are distributed in a skewness fashion.

As mentioned, the points of interest (POI) of domain applications are usually within a certain sub-region for a certain time duration, while evolving to neighboring sub-regions moderately. Queries are often issued periodically and continuously; a query region is typically part of a POI region, and concurrent queries often have overlapping sub-regions. Experiments are conducted for SAQSR, where a skewness degree is set to 60% and the cache size is set to 600. A POI region is assumed to be a rectangle in shape and is set to be dense or sparse sub-regions of the network. A query region is encoded as a set of grid cells contained in the POI region. These grid cells may not be neighboring, for simulating the scenario where multiple queries are issued concurrently. Grid cells at a certain time slot are randomly determined, and the number of grid cells may be different in contiguous time slots. The settings of experiments include: (i) *S1-sparse*: sparse sub-regions with our cache mechanism; (ii) *S2-sparse*: sparse sub-regions without our cache mechanism; (iii) *S1-dense*: dense sub-regions with our cache mechanism; and (iv) *S2-dense*: dense sub-regions without our cache mechanism. Figure 7 shows the energy consumption for these four types of experimental configurations. Intuitively, our cache mechanism can reduce the energy consumption to a large extent, when the query region is within sparse or dense sub-regions, *i.e.*, *S2-sparse* is 348% more in energy consumption than *S1-sparse*, and *S2-dense* is 599% more in energy consumption than *S1-dense*. Note that the difference of energy consumption for *S1-sparse* and *S1-dense* is quite small. This indicates that our cache mechanism is efficient in reducing energy consumption and increasing the network capability, especially when the cache of the SN can accommodate almost all sensory data interested in queries.

**Figure 10:**
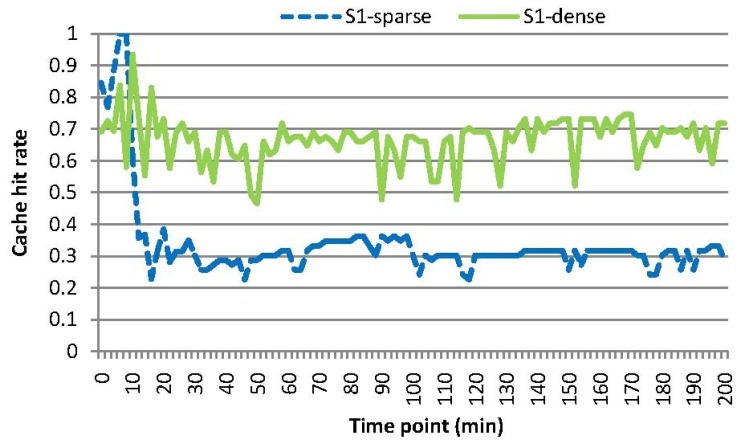
Comparison of cache hit rates for *S1*-*sparse* and *S1*-*dense*, where *S1*-*sparse* means sparse sub-regions with our cache mechanism, and *S1-dense* means dense sub-regions with our cache mechanism. Similar to Figure 6, this figure shows that our cooperative caching mechanism benefits the cache hit rates for cached sensory data of the SN (roughly from 30%–65%), especially when sensors nodes are densely deployed in the network.

Figure 10 shows the cache hit rates for the scenarios *S1-sparse* and *S1-dense*. Generally, the cache hit rate of *S1-sparse* (roughly 30%) is lower than that of *S1-dense* (roughly 65%), although the energy consumption for *S1-sparse* and *S1-dense* is almost the same, as shown in Figure 7. Since grid cells are chosen randomly for representing a query region, common grid cells between continuous queries are relatively less in number than those of Figure 6, which induces a smaller value of the cache hit rates. Note that relatively few sensor nodes are involved in *S1-sparse*, and a minor change of the number of sensor nodes may have a relatively big impact on cache hit rates, which results in a relatively smaller value of cache hit rates for *S1-sparse*. Consequently, our cache mechanism is more efficient, especially when periodic and continuous queries have more overlapping sub-regions.

### 5.3. Comparison with Relevant Techniques

This section presents the results of our experiments comparing the efficiency and performance of our popularity-based cooperative caching mechanism (denoted PCC) with respect to those of our multiple-attribute query processing (MQP) mechanism, as presented by Zhou *et al.* [33]. Different from PCC, the cooperative caching mechanism has not been adopted in MQP for facilitating the query processing with the same environmental settings.

**Figure 11:**
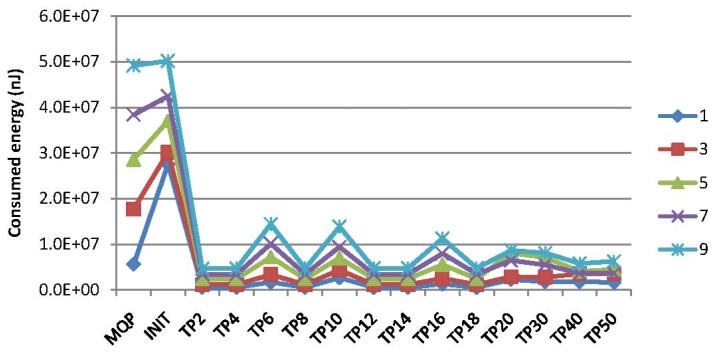
Comparison of energy consumption of our popularity-based cooperative caching (PCC) and multiple-attribute query processing (MQP) [33] for the query types of MAQWR and SAQWR, where the number of attributes is set to 1, 3, 5, 7 and 9, respectively. The energy consumption at various time points (TP2, TP4, *etc.*) are illustrated. Generally, the energy consumption of our PCC is much smaller than that of MQP (roughly 30% less on average for PCC than MQP), especially when the number of query attributes is relatively large. Note that the energy consumption of PCC is quite large at the first time point, since no sensory data have been cached in the SN for reducing the data gathering from the network in real-time to facilitate the query processing.

Experiments have been conducted for the comparison of the energy consumption for PCC and MQP [33] with respect to the query types of MAQWR and SAQWR. The number of attributes are set to 1, 3, 5, 7 and 9, respectively. The cache size of the SN is set to 900, and the skewness degree is set to 60%. Figure 11 shows the experimental results, where the energy consumption at certain time points (e.g., TP2, TP4, *etc.*) is illustrated. Note that the symbol *TPi* (e.g., *i* = 2) means the *i*-th (second) time point. It is worth mentioning that the energy consumption for MQP is almost the same for all time points, whose values are illustrated at the left side of Figure 11. As for our PCC, the energy consumption is quite large at the first time point (denoted INITin Figure 11), since no sensory data have been cached in the SN for reducing the data gathering from the network in real time, and all intermediate nodes (INs) are required to gather sensory data from the corresponding sensor nodes and to cache them locally. The energy consumption at the succeeding time points decreases to a large extent due to the reusability of sensory data cached in the SN for supporting the forthcoming query answering. This figure shows that the energy to be consumed will be in a steady state after around 18 time slots when the number of attributes is one and around 40 time slots when the number of attributes is nine, due to the fact that sensory data cached in the SN and INs can hardly reduce the energy consumed for query processing any further. Generally, our PCC outperforms MQP on energy consumption, especially when the query attributes are relatively large in number.

**Figure 12:**
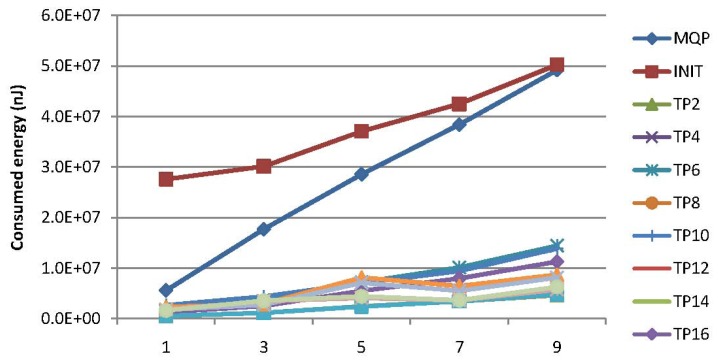
Comparison of energy consumption of our PCC and MQP [33] for the query types of MAQWR and SAQWR, where the number of attributes are set to 1, 3, 5, 7 and 9, respectively. This figure shows that more energy is consumed for our PCC than MQP at the first time point (denoted INIT), while much less energy is consumed afterwards.

Figure 12 illustrates the energy consumption for our PCC and MQP [33] at different time points. As previously mentioned, the energy consumption for MQP is the same for all time points. Figure 12 shows that more energy is consumed at the first time point (denoted INIT). The energy consumption for our PCC is much less than that of MQP in the consequent time points. It is evident from this figure that our PCC is more energy efficient than MQP, especially when the query attributes are relatively large in number.

## 6. Related Work and Comparison

Traditional techniques have been developed for facilitating the query processing in wireless sensor networks (WSNs) leveraging the cooperative caching mechanism. We have proposed a popularity-based caching mechanism for optimizing periodic queries in WSNs [42]. One kind of attribute is sensed by sensor nodes in the network. Sensory data are cached only in the memory space of the sink node, and these data are assumed to be valid for answering the forthcoming queries within a certain number of time slots. Usually, the point of interest evolves moderately to neighboring sub-regions, whose sensory data may have become stale already and are not being cached at the sink node at this moment. To facilitate this query processing procedure, grid cells, which may be covered by the forthcoming queries, are derived from the previous queries according to the popularity of interested grid cells. Sensory data are pre-fetched from the network for these grid cells whose popularity is among the highest. Generally, the technique developed in this article is inspired by our previous work [42]. However, multiple kinds of attributes are considered to be sensed by sensor nodes in this technique, and the staleness of sensory data is determined according to whether sensory data have been changed significantly. Besides, this technique removes the assumption made by Zhou *et al.* [42] that the sink node has enough storage capability for caching sensory data of sensor nodes in the whole network. Instead, only sensory data, which have a high possibility of being reused for answering the forthcoming queries for certain attributes, are cached in the sink node. Grid cells, which are not covered by recent queries, cache sensory data of certain attributes or just a flag indicating a dramatic change of sensor nodes. This two-tiered cooperative mechanism, which caches sensory data at the sink node and the head nodes of grid cells, is efficient for facilitating the query answering, as evidenced by the experimental evaluation in Section 5.3.

A cluster-based cooperative caching mechanism is developed by Chauhan *et al.* [47] for supporting query processing. The network is divided into non-overlapping clusters, and each sensor node is assumed to have some cache space. Sensory data are stored in the cache space of sensor nodes that are near the sink node. When a query request is to be responded to, sensory data are retrieved through a cache discovery process. Generally, a sensor node that is responsible for this query is examined for determining whether the required sensory data are saved in its cache space. If not, the cluster of the source sensor node is examined, and the source sensor node is visited for routing the required sensory data to the sink node. This method claims to reduce the requirement for bandwidth, energy and storage of the network. However, the sink node is not responsible for caching sensory data. In fact, sensor nodes near the sink node should cache sensory data and are responsible for routing data to the sink node. These sensor nodes have much more energy consumption and should deplete their energy quickly. In our technique, sensory data are cached in the sink node and the head nodes of grid cells, and the popularity of sensor nodes is considered when determining which sensory data of certain attributes should be cached. A cooperative caching mechanism is developed by Sharma *et al.* [48], where sensory data are cached in the sink node and sensor nodes. A cache zone is formed as a region around a sensor node, where the storage of surrounding sensor nodes can be used to build a larger cumulative cache. A cache discovery mechanism is proposed for identifying required data items, and a cache replacement policy is developed for evicting data items of less importance. This is an interesting work and inspired us to develop our technique. Generally, this work tries to increase data availability nearer the sink and to reduce unnecessary energy consumption. A query processing mechanism is not discussed specifically when multiple queries are issued periodically and continuously.

To better support the cooperative caching in WSNs, sensor nodes, which can take the role of coordinating and packet caching and forwarding, are essential. A metric of energy betweenness centrality is proposed by Dimokas *et al.* [49] to evaluate the significance of sensor nodes and to examine whether these sensor nodes can take the special role of cooperation concerning the caching decisions. Consequently, a new energy-efficient cooperative caching protocol is developed. An in-network distributed query processor called Corona is developed by Khoury *et al.* [32], which aims to cluster sensor readings into a local storage buffer in sensor nodes. When the freshness of these sensory data is within a certain threshold, they can be used for answering concurrent queries directly, rather than being fetched from the network in real time. Therefore, sensor activation can be minimized. A survey is presented by Kumar *et al.* [30] about cache-based policies in WSNs for reducing the network traffic and bandwidth usage. Besides, cooperative caching has been used in other domains, like mobile *ad hoc* networks, to increase data availability and reduce data access delays [50], and cooperative caching policies have been applied in social wireless networks for minimizing electronic content provisioning cost [51]. Generally, these techniques explore the routing of data packets in the network, and a single query processing is of interest mostly. The strategy of caching sensory data in the intermediate nodes is the main focus; whereas in this article, we propose a two-tiered cooperative caching mechanism for reducing the energy consumption of query answering in the forthcoming time slots.

To establish efficient paths for routing sensory data to the sink node, a cache-based routing metrics is proposed by Grilo *et al.* [29], where intermediate nodes in WSNs are used for caching packets and transmitting them to the sink node. Note that in a heterogeneous Internet of Things, sensor nodes may differ to some extent in terms of storage and computational capabilities. Hence, sensor nodes with larger capabilities are good candidates for caching the packets. Therefore, cache utilization is set as a novel metric applied in this technique, and routing paths should be selected considering cache-rich intermediate nodes. Generally, this work is interesting and inspires caching packets at the intermediate nodes and determining appropriate routing paths; whereas we explore a cooperative caching mechanism at the sink node and the head nodes of grid cells, according to the popularity of sensory data leveraging the recent query history. These two techniques can complement each other for better facilitating the query processing and, thus, improving the energy efficiency. A caching platform is presented by Léone *et al.* [52] for reducing the network communication cost. A gateway based on a constrained application protocol (CoAP)-HTTP proxy is proposed, where cross-layer data are cached in the proxy [53]. When a query is to be answered, the gateway will delegate for query answering. Only when the requested sensory data are not fresh enough or missed in the cache, this query should be transferred to the network for fetching data. This work is similar to what we have developed. However, mainly a caching model is presented, while technical details about the caching strategy are not clear.

Besides, some methods study periodic query processing. In [17,24], the authors study the achievable network capability of snapshot and continuous data collection for a probabilistic WSN model. Cell-based path scheduling and zone-based pipeline scheduling algorithms are proposed for improving the concurrency of snapshot and continuous data collection, respectively. This work inspired us to partition the network region into square grid cells and to use grid cells as elementary units for caching sensory data. Node localization is an important research problem, especially for large-scale WSNs [54,55]. The network is divided into overlapped local networks, and the corresponding local maps are constructed using a local semidefinite programming method. These local maps are merged into a global map, which contains the exact position of the nodes of interest. It is argued that in certain WSN applications, query requests come periodically with stringent delay constraints [10]. Therefore, a periodic aggregation query scheduling is performed by the designed routing strategy along with packet scheduling protocols. The quality of queries in WSNs is discussed by Brayner *et al.* [56], which aims to deliver a reasonable level of data quality as expected, while ensuring the intelligent consumption of limited network resources. Generally, these methods mainly explore the strategies of supporting (periodic) query processing in WSNs, in order to consume less resources while prolonging the network lifetime. The sharing of sensory data retrieved at a certain time slot and between concurrent queries and the reuse of sensory data gathered by recent queries for answering the forthcoming queries are not discussed extensively.

## 7. Conclusions

Wireless sensor networks, which act as important interfaces between physical environments and computational systems, have been used extensively to support widespread application domains. Usually, multiple attributes should be sensed in a network, and multiple-attribute sensory data are queried from the network continuously and periodically for facilitating domain applications. Note that sensory data may not change significantly within a certain time duration, and applications may tolerate a variation of adopted sensory data with accurate ones to a certain extent. Consequently, sensory data gathered at this moment can be shared for answering concurrent queries and may be reused for answering the forthcoming queries. To remedy this issue, a two-tier cooperative caching mechanism is proposed in this article. Specifically, the popularity of sensory data, which reflects the possibility of reusing these data by the forthcoming queries, is calculated according to the queries issued in recent time slots. Sensory data of a higher popularity are cached in the sink node, and these data can be used for query answering directly. Sensory data of a lower popularity are cached in the head nodes of grid cells. This two-tier cooperative caching strategy promotes the reuse of sensory data for answering the forthcoming queries significantly. The results of experimental evaluation show that our technique is efficient in the reduction of energy consumption for query answering, especially when the number of queries is relatively large. Specifically, the energy consumption for the case when the sink node is lacking caching space is around 40%∼66% more, as shown in Figure 5, than that for the case when the sink node is capable of caching all sensory data requested by the queries, while the cache hit rate increases significantly, as well (roughly from 70%–95%, as shown in Figures 8 and 9) for these two cases. Compared with our previous technique [33], this two-tier cooperative caching strategy can reduce around 30% of the energy consumption for answering the queries, as shown in Figure 11.

As to the future directions, we are adopting this cooperative caching mechanism to the scenario where a wireless sensor network is shared by multiple applications. The challenge includes the sharing and reusing of query results of these applications for answering forthcoming queries, in order to reduce the energy consumption. Besides, sensory data pre-fetching from the network should be beneficial for the decrease of energy consumption, especially when the forthcoming queries can be (partially) predicted according to the queries in the past. A prediction model is under the construction.

## Figures and Tables

**Table 1:** Parameters in the energy model [34]

**Name**	**Description**
*E_elec_*	Energy consumption constant of the transmitterand receiver electronics
*ε_amp_*	Energy consumption constant of the transmission amplifier
*k*	The number of bits in one pocket
*d*	The distance of transmission
*n*	The attenuation index of transmission
*r*	The communication radius of sensor nodes
*E_Tx_*(*k*, *d*)	The energy consumed to transmit a *k* bit packet to a distance *d*
*E_Rx_*(*k*)	The energy consumed to receive a *k* bit packet
*E_ij_*(*k*)	Energy consumption for transmitting a *k* bit packet from a node *i* to a neighboring node *j*

**Table 2:** An example of a bitmap for attributes interested in a certain query *q*, where *atr_i_* specifies the *i*-th attribute. The value 1 means that a certain attribute (e.g., *atr*_3_) is interested in this query *q*, while 0 means it is not.

**Attribute**	*atr*_1_	*atr*_2_	*atr*_3_	*atr*_4_	…	*atr*_k_

**Bit**	1	0	1	0	…	0

**Table 3:** An example of the GC-attribute table, where *gc_i_* specifies the *i*-th grid cell and *atr_j_* specifies the *j*-th attribute. The value 1 means that the sensor nodes in a certain grid cell (e.g., *gc*_1_) are interested in a certain attribute (e.g., *atr*_3_), while 0 means they are not.

	*gc*_0_	*gc*_1_	*gc*_2_	*gc*_5_	*gc*_6_	*gc*_7_
*atr*_1_	0	1	0	0	1	1
*atr*_2_	0	0	0	0	0	0
*atr*_3_	1	1	1	1	1	0
…	…	…	…	…	…	…
*atr_k_*	0	0	0	0	0	0

**Table 4:** An example of the GC-SNCache table, where *gc*_i_ specifies the *i*-th grid cell and *atr*_j_ specifies the *j*-th attribute. The value 1 means that sensory data for a certain grid cell (e.g., *gc*_1_) with a certain attribute (e.g., *atr*_1_) are cached in the memory of the SN, while 0 means they are not.

	*gc*_0_	*gc*_1_	*gc*_2_	*gc*_5_	*gc*_6_	*gc*_7_
*atr*_1_	1	1	0	0	1	0
…	…	…	…	…	…	…
*atr*_i_	0	1	0	1	1	0
…	…	…	…	…	…	…
*atr_k_*	0	1	0	1	0	1

**Table 5:** Parameters settings in the experiments.

**Parameter Name**	**Value**
Network region	350 m × 350 m
Skewness degree	20%–80%
Number of sensor nodes	1000
Number of attributes	10
Cache size in the SN	500–900
Communication radius (r)	50 m
Attenuation index of transmission (n)	2
Energy consumption constant for the transmit and receiver electronics (*E_elec_*)	50 nJ/bit
Energy consumption constant for the transmit amplifier (*ε_amp_*)	100 pJ/(bit × m^2^)
Attenuation coefficient (*α*)	0.6
Number of time slots considered for computing the popularity of vectors (*k*)	4
Length of a time slot for query processing	2 min
Time interval for data synchronization	5 min
Time interval for head node reselection	20 min
